# Impact of different blood group incompatibilities in kidney transplantation: a 15-year outcomes analysis from a large kidney transplant center

**DOI:** 10.3389/frtra.2025.1690999

**Published:** 2025-11-18

**Authors:** Dalia A. Obeid, Dieter C. Broering, Khalid A. AlMeshari, Yaser Z. Shah, Hassan A. Aleid, Hadeel M. AlManea, Amira M. AlAbassi, Nour AlMozain, Kris Marquez, Eman A. Alsaadi, Tariq Z. Ali

**Affiliations:** 1 Transplant Research and Innovation Department, Organ Transplant Centre of Excellence, King Faisal Specialist Hospital and Research Centre, Riyadh, Saudi Arabia; 2 Department of Kidney and Pancreas Transplantation, King Faisal Specialist Hospital and Research Centre, Riyadh, Saudi Arabia; 3 Department of Pathology and Laboratory Medicine, King Faisal Specialist Hospital and Research Center, Riyadh, Saudi Arabia; 4 Abdominal Transplant & Hepatobiliary Surgery Centre Department, Organ Transplant Centre of Excellence, King Faisal Specialist Hospital and Research Centre, Riyadh, Saudi Arabia

**Keywords:** ABO-incompatible transplantation, antibody-mediated rejection, kidney transplant outcomes, long-term survival, AMR

## Abstract

**Background:**

While ABO-incompatible kidney transplantation (ABOiKT) has demonstrated favorable short-term outcomes, data on its long-term effects remain limited. This study evaluated the short- and long-term clinical outcomes of ABOiKT across various ABO-incompatible donor–recipient combinations.

**Methods:**

We included patients who underwent ABOiKT at our institution in 2007–2024. The outcomes assessed included 15-year data on graft, patient survival, and early AMR rates.

**Results:**

Of 239 ABOiKT cases, AMR occurred in 9.2% and was linked to longer hospitalization and higher graft failure. AMR was most frequent in B–O (20.3%) and A1–O (13.3%) transplants but no cases of AMR were observed in the recipients of kidneys from A2 donors. B to O mismatch significantly increased the risk of AMR-related graft loss. Patient survival was 99.1% at 1 year and 86.2% at 15 years and Graft survival was 92.7% and 87.5% respectively.

**Conclusions:**

Our study showed favorable outcomes of ABOiKT across different mismatch types. As the largest ABOiKT study in the Middle East with extended follow-up, our study provides important regional insights and contribute significantly to the global understanding of ABOiKT outcomes.

## 1 Introduction

Given the continuing worldwide shortage of kidney donors, alternative strategies to expand the donor pool must be established. ABO-incompatible kidney transplantation (ABOiKT) has emerged as a feasible option for patients with end-stage renal disease (ESRD). The first series of successful ABOiKTs was reported in Belgium in 1981, which involved 26 patients who underwent plasmapheresis and splenectomy and achieved a 1-year survival rate of 75% (1). These pioneering efforts demonstrated the viability of overcoming immunological barriers and laid the foundation for future advancements in ABOiKT.

Subsequent advances have significantly improved ABOiKT outcomes, particularly the introduction of plasmapheresis, antigen-specific immunoadsorption, and B-cell depletion therapies, such as rituximab for suppressing antibody production. Several recent studies (2) have reported 1-year survival rates exceeding 95%. These developments have not only increased the number of available kidney donors by overcoming differences between blood groups and human leukocyte antigen (HLA) incompatibilities, they have also established new standards for the immunosuppressive management of kidney transplantation.

Since the early success of ABOiKT, numerous studies have evaluated the short- and long-term recipient outcomes. While earlier reports have obtained poor outcomes for ABO-incompatible (ABO-i) recipients compared with ABO-compatible (ABO-c) recipients, these have significantly improved in more recent efforts. The gap in graft survival between ABO-c and ABO-i transplants has narrowed over time, especially in centers with established protocols (3–6). Optimizing desensitization and maintenance immunosuppression strategies are crucial for enhancing patient and graft outcomes. A longitudinal study attributed significant improvements over time in graft survival rates in ABOiKT to optimized protocols (7). Moreover, some centers have observed fewer infectious complications following adjustments to immunosuppressive strategies (8).

Despite this progress, ABOiKT remains significantly challenging in clinical practice. Recipients still have an increased risk of antibody-mediated rejection (AMR), graft loss, and infectious complications, primarily because of the intensified immunosuppressive regimens required to prevent organ rejection (9). A recent meta-analysis (10) found significant differences between ABO-i and ABO-c transplants in terms of graft survival (96% vs. 98%), infectious causes of death (49% vs. 13%), and AMR rates (12% vs. 6%). Although these findings indicate highly improved ABOiKT outcomes, the risk of adverse effects remains high (11).

Our institution is recognized as the largest organ transplantation center in the Middle East. To date, we have performed over 5,000 kidney transplants. In 2024 alone, 511 kidney transplants were performed at our center, reflecting the high volume of renal transplant activity managed annually. The ABOiKT program was initiated at our center in 2007. Our preliminary ABOiKT protocol yielded favorable short-term outcomes, as evidenced by 1-year patient and graft survival rates of 100% and 94.8%, respectively, along with a low incidence of infectious complications (12). Nevertheless, our understanding of the mechanisms underlying the rejection and optimization of immunosuppressive regimens remains significantly incomplete. Moreover, the specific effects of various donor-recipient ABO incompatibility patterns have been insufficiently explored. In this study, we aimed to assess the impact of different ABO mismatches on transplantation outcomes, including both short-term results and long-term follow-up extending up to 15 years.

## 2 Materials and methods

### 2.1 Study population and data collection

This study included adult patients (aged >18 years) who underwent kidney transplantation from ABO-incompatible donors at a specialized hospital in Riyadh, Saudi Arabia, between 2007 and 2024. Clinical and demographic data were collected from the hospital records. The Ethics Committee of our institution approved the use of data from the kidney transplant registry for noninterventional research studies (RAC 2121012). The study was conducted in accordance with the principles of the Declaration of Helsinki and the Declaration of Istanbul. Details of the desensitization protocol used for ABOi-KT at our center are described in the Supplementary Appendix.

### 2.2 Statistical analysis

All collected data were stored and analyzed using R version 4.3.0 (R Core Team, Vienna, Austria, 2025). Inferential and descriptive statistics were performed to assess the patients' demographic and clinical characteristics. Numerical variables were assessed using the Wilcoxon rank-sum test. Differences among groups for categorical variables were analyzed using the chi-square test or Fisher's exact test and reported as frequencies (percentages).

The risk of AMR was estimated using binary firth's penalized logistic regression models and expressed as odds ratios (OR). Survival analysis was performed using the Cox proportional hazards regression univariate model to calculate hazard ratios (HRs) of graft dysfunction. Kaplan–Meier plots and log-rank tests were used to analyze the differences between the survival curves. Overall survival was measured from the date of transplantation to the date of the clinical event (AMR/graft loss). Statistical significance was set at *p* < 0.05, and all interval estimates were calculated using 95% confidence intervals (CIs).

## 3 Results

### 3.1 Patients' clinical and demographical characteristics by AMR status

We performed 239 ABOiKT procedures over a 15-year follow-up period. The study cohort had a mean age of 43.4 years, with almost equal sex distribution (51.5% were male). The underlying cause of kidney disease was unknown in 40.2% of the ABOiKT recipients. Most transplants were performed using related donors (68.6%) and were characterized by low HLA mismatch (0–4 mismatches in 80%) and negative flow crossmatch (84%). Among the donors, the most common blood types were B (37.0%) and A2 (36.0%), whereas most recipients had an O blood type (73%). At the end of the follow-up period, the overall mortality rate in our cohort was 4.2% (*n* = 10), with graft failure occurring in 9.2% of patients (*n* = 22).

The cohort characteristics stratified by post-transplant AMR status is summarized in Table 1. AMR occurred in 22 patients (9.2%), with a mean onset of 10.5 days after transplantation. AMR was significantly associated with donor ABO blood group (*P*-value < 0.001) and ABO incompatibility (*p* < 0.001). Early graft loss due to AMR occurred in 14 patients within 30 days post-transplantation. Furthermore, graft loss unrelated to AMR over the long term occurred in 8 patients, with causes including noncompliance (*n* = 2), multiple episodes of acute cellular rejection (*n* = 1), and disease recurrence (*n* = 5).

**Table 1:** Patients’ clinical and demographical characteristics by AMR status.

Characteristics	*N*	Overall	No AMR	AMR	*p*-value**
		*n* = 239*	*n* = 217*	*n* = 22*	
Recipient's sex	239				0.89
Female		116 (48.5%)	105 (90.5%)	11 (9.5%)	
Male		123 (51.5%)	112 (91.1%)	11 (8.9%)	
Recipient's age	239	43.36 (15.57)	43.41 (15.67)	42.82 (14.90)	0.94
Causes of ESRD	239				0.61
Diabetes mellitus type 1		9 (3.8%)	8 (88.9%)	1 (11.1%)	
Diabetes mellitus type 2		36 (15.1%)	35 (97.2%)	1 (2.8%)	
FSGS		16 (6.7%)	14 (87.5%)	2 (12.5%)	
GN		44 (18.4%)	40 (90.9%)	4 (9.1%)	
HTN		10 (4.2%)	10 (100.0%)	0 (0.0%)	
Other		19 (7.9%)	16 (84.2%)	3 (15.8%)	
Unknown		96 (40.2%)	85 (88.5%)	11 (11.5%)	
Urology		9 (3.8%)	9 (100.0%)	0 (0.0%)	
Relationship categories	239				0.39
Child		60 (25.1%)	57 (95.0%)	3 (5.0%)	
Non-related, PKE		75 (31.4%)	69 (92.0%)	6 (8.0%)	
Other relatives		18 (7.5%)	15 (83.3%)	3 (16.7%)	
Parent		9 (3.8%)	9 (100.0%)	0 (0.0%)	
Sibling		67 (28.0%)	58 (86.6%)	9 (13.4%)	
Spouse		10 (4.2%)	9 (90.0%)	1 (10.0%)	
HLA Mismatch Categories	239				0.78
>4		47 (19.7%)	42 (89.4%)	5 (10.6%)	
0–4		192 (80.3%)	175 (91.1%)	17 (8.9%)	
Length of Stay (Days)	239	17.92 (7.54)	17.35 (7.04)	23.55 (9.94)	<0.001*
Incompatibility	239				<0.001*
A1 to B		19 (7.9%)	18 (94.7%)	1 (5.3%)	
A1 to O		45 (18.8%)	39 (86.7%)	6 (13.3%)	
A1B to O		1 (0.4%)	0 (0.0%)	1 (100.0%)	
A2 to B		16 (6.7%)	16 (100.0%)	0 (0.0%)	
A2 to O		69 (28.9%)	69 (100.0%)	0 (0.0%)	
B to A		30 (12.6%)	28 (93.3%)	2 (6.7%)	
B to O		59 (24.7%)	47 (79.7%)	12 (20.3%)	
Cross-match	239				>0.99
B+/T+		16 (6.7%)	15 (93.8%)	1 (6.3%)	
Negative		204 (85.4%)	184 (90.2%)	20 (9.8%)	
Only B+		19 (7.9%)	18 (94.7%)	1 (5.3%)	
Donor's sex	239				0.79
Female		52 (21.8%)	48 (92.3%)	4 (7.7%)	
Male		187 (78.2%)	169 (90.4%)	18 (9.6%)	
Donor's age	239	31.85 (8.15)	31.79 (8.10)	32.36 (8.84)	0.70
DGF	239	24 (10.0%)	17 (70.8%)	7 (29.2%)	0.003*
No of sessions pre-Tx	236	2.51 (1.58)	2.48 (1.61)	2.81 (1.21)	0.4
No. of sessions post-Tx	238	1.57 (2.18)	1.41 (2.13)	3.09 (2.16)	<0.001*
Graft failure	239	22 (9.2%)	8 (36.4%)	14 (63.6%)	<0.001*
The patient died	233	10 (4.2%)	8 (80.0%)	2 (20.0%)	0.2

AMR, antibody-mediated rejection; ESRD, end-stage renal disease; FSGS, focal segmental glomerulosclerosis; GN, glomerulonephritis; HTN, hypertension; PKE, paired kidney exchange; DGF, delayed graft function; Tx, transplantation.

* *n* (%); Mean (standard deviation).

** Pearson's chi-squared test; Wilcoxon rank sum test; Fisher's exact test.

**Figure 1:**
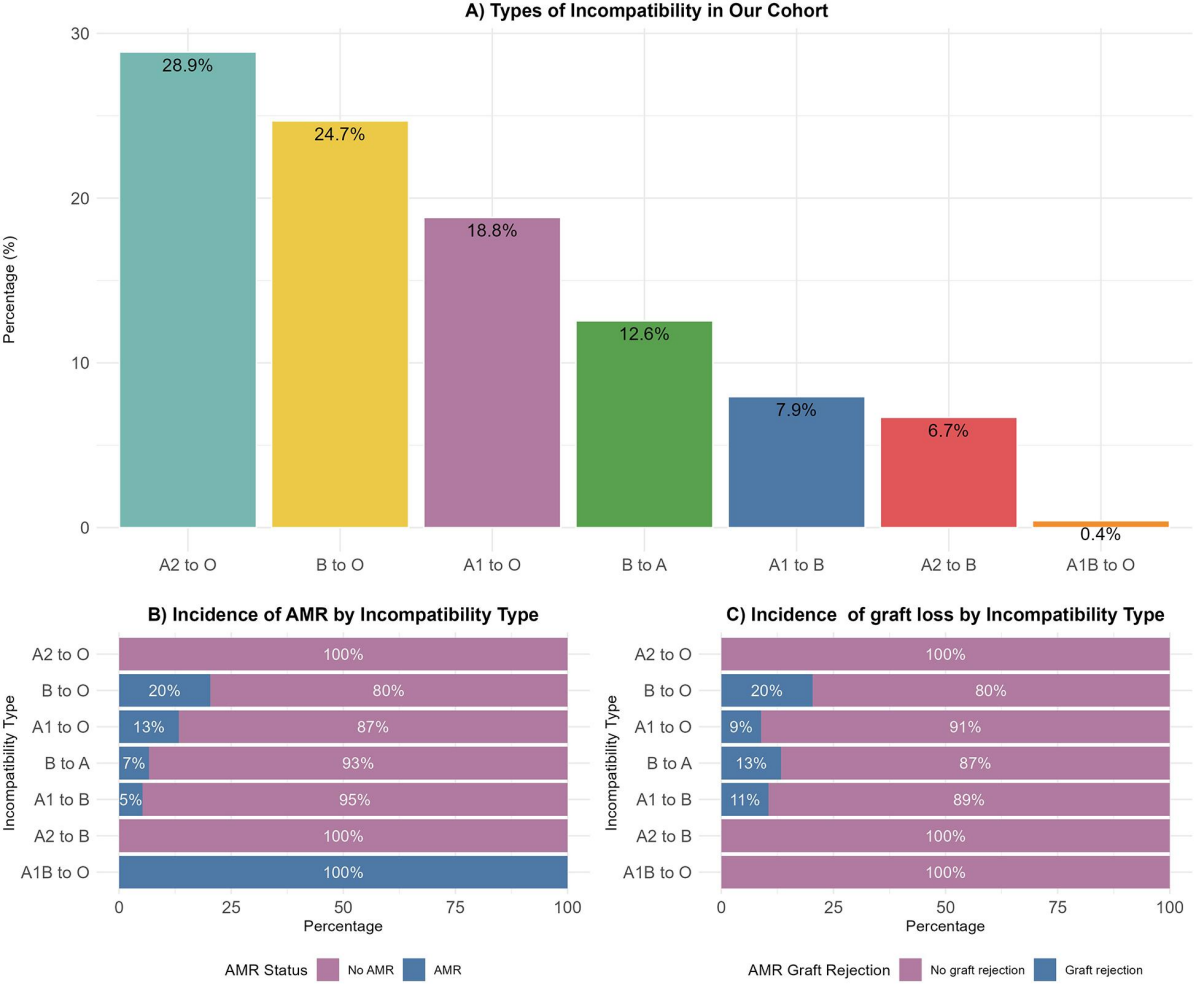
**(A)** Distribution of ABO-incompatible donor-to-recipient pairs according to the incompatibility type. **(B)** Proportion of patients with AMR within each type of incompatibility. **(C)** Proportion of patients with graft loss within each incompatibility type. The bars represent the percentage values.

Among the seven ABOi mismatches, the most common incompatibility was A2 to O (28.9%), followed by B to O (24.7%) and A1 to O (18.8%) (Figure 1). When stratified by AMR, the B to O and A1 to O incompatibilities had the highest rates of AMR at 20% and 13%, respectively (Figure 1). No AMR events were observed in A2 to O or A2 to B transplants. Furthermore, AMR was successfully reversed in 8 patients, most of whom maintained graft function and half of which involved B to O transplants (*n* = 4). Interestingly, graft failure occurred mostly with B to O incompatibility (20%); however, no AMR related graft losses were occurred in transplants from A2 and A1B donors (Figure 1).

**Figure 2:**
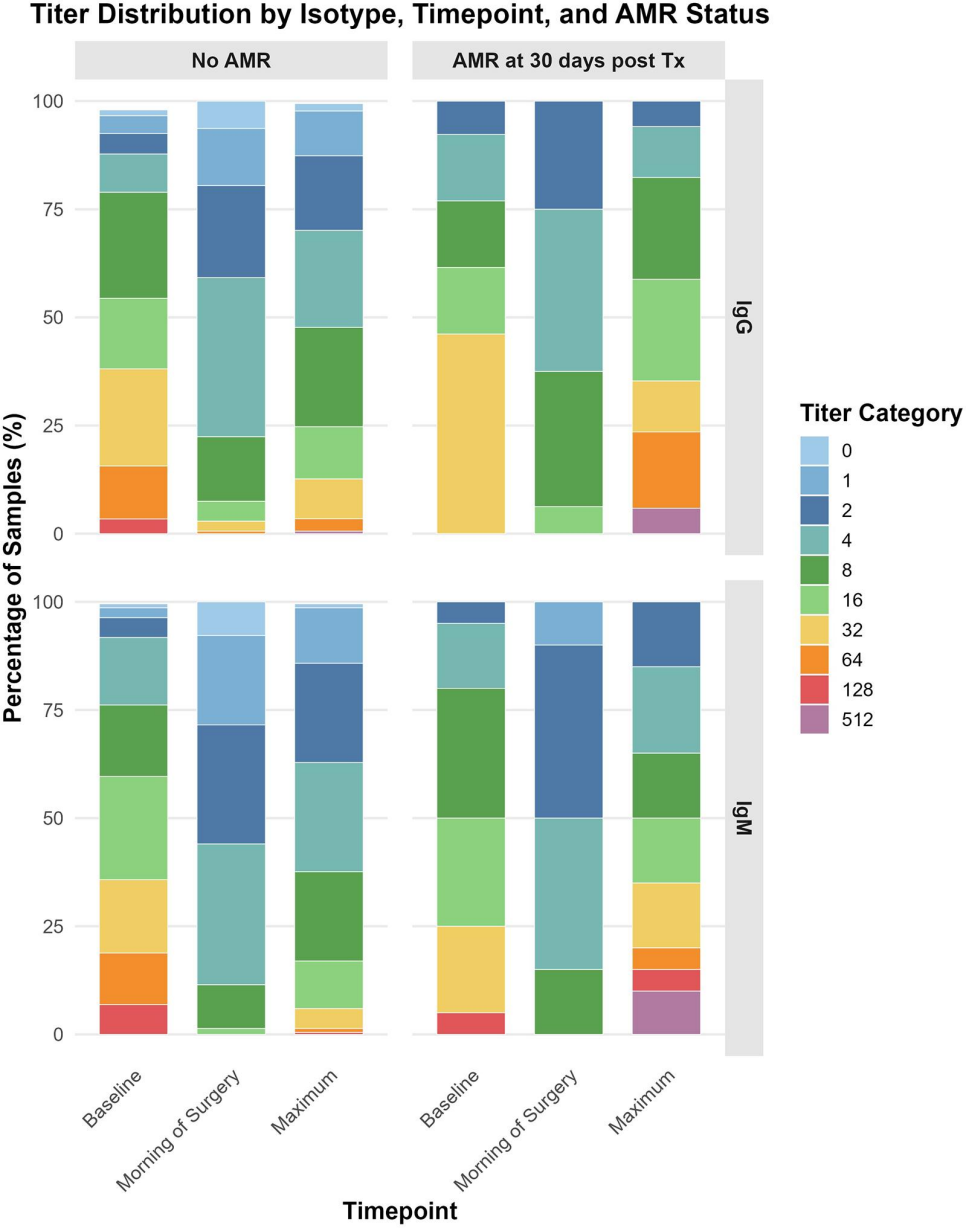
Isohemagglutinin (immunoglobulin [Ig]G and IgM) titers at baseline, on the morning of surgery, and at their post-transplant peak (1–14 days post-transplantation), stratified by AMR status and analyzed by titer category. Titers were categorized for interpretability as follows: 0–1, undetectable or too low to quantify; 2–4, very low; 8–16, low to moderate; 32–64, moderate to high; 128–256, high; and 512, very high (maximum level detected).

Figure 2; Supplementary Table 1 show isohemagglutinin (IgG and IgM) titers at baseline, surgery day, and post-transplant peak, stratified by AMR status. No significant differences in IgG or IgM titers existed between AMR and non-AMR groups at baseline and surgery. However, maximum posttransplant titers were significantly higher in AMR patients, with 17.6% reaching IgG titer of 64 vs. 3.3% in non-AMR (*p* = 0.035). More AMR patients had IgM titers ≥ 32, with 15% reaching 32% and 10% reaching 512, compared to 5.3% and 0% in non-AMR (*p* = 0.003).

**Figure 3:**
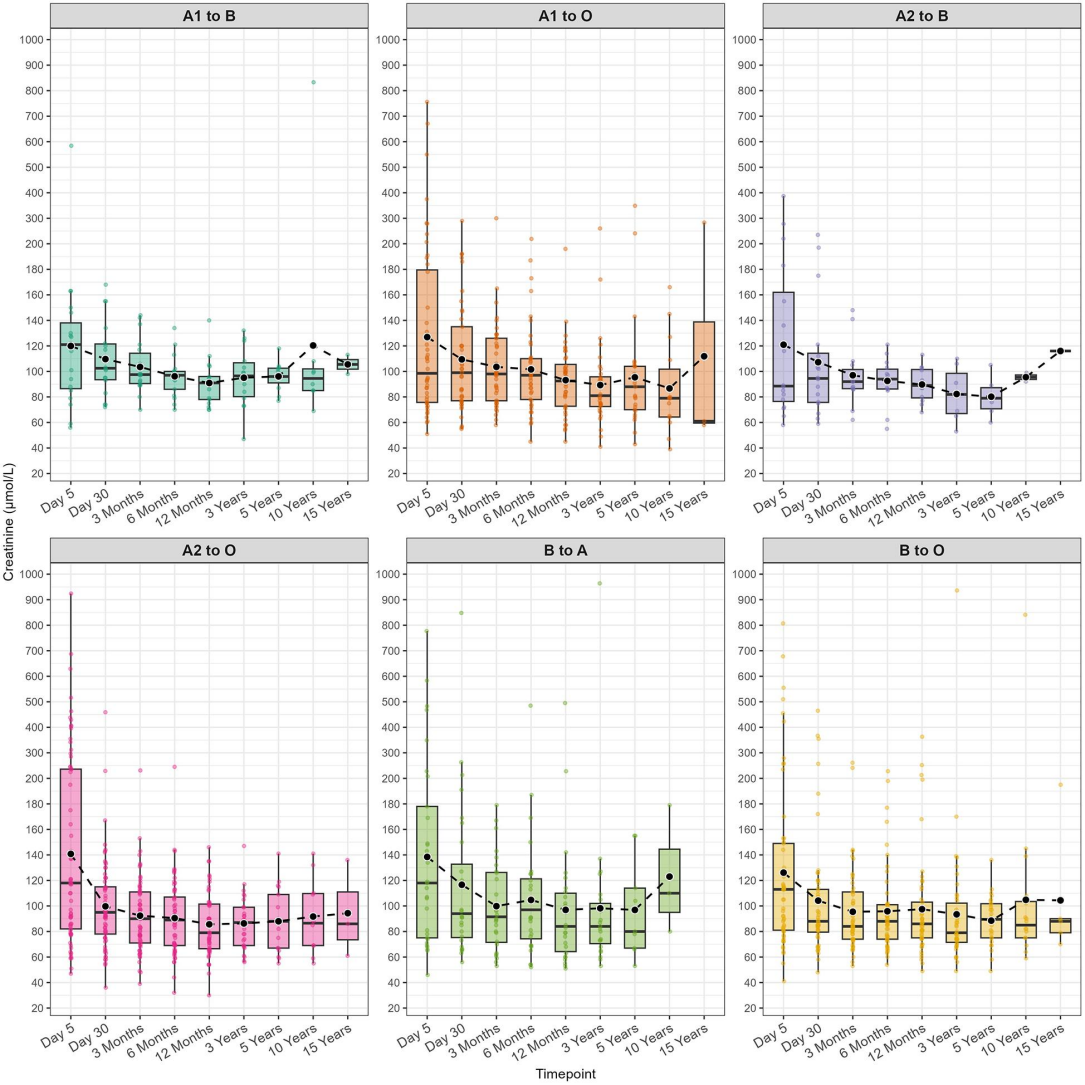
Bar graphs for serum creatinine (μmol/L) levels at nine time points across different ABO mismatch groups.

The median serum creatinine levels were also assessed at different time points across the ABO mismatch groups (Figure 3; Supplementary Table 2). The kidney function remained excellent across all ABO mismatch combinations.

### 3.2 Clinical and demographical predictors of AMR

Table 2 presents the results of the univariate and multivariate logistic regression analysis of risk factors for AMR and graft loss. Most baseline demographic and clinical characteristics were not significantly associated with AMR. Among incompatibility groups, B to O incompatibility exhibited four times the risk of AMR compared with A1/2 to O incompatibility groups (OR 4.39, 95% CI: 1.65–12.8; *p* = 0.003).

**Table 2:** Univariate and multivariate binary logistic model analysis for predicting AMR based on patient clinical and demographic data.

Characteristic	Univariate AMR (Firth)		Multivariate AMR (Firth)	
	OR (95% CI) *^a^*	*P*	aOR (95% CI)^a^	*P*
Recipient sex	0.94 (0.39–2.24)	0.88	–	–
Recipient age (per year)	1.00 (0.97–1.03)	0.87	–	–
ESRD				
GN/FSGS vs. DM	2.08 (0.50–11.7)	0.32	–	–
Others vs. DM	1.72 (0.32 to 10.8)	0.53	–	–
Unknown vs. DM	2.34 (0.65–12.4)	0.21	–	–
Incompatibility category *^b^*				
Other ABO incompatibility vs. Anti-O incompatibility ^b^	1.20 (0.32–4.12)	0.77	2.45 (0.49–12.3)	0.26
B to O incompatibility vs. Anti-O Incompatibility ^b^	4.39 (1.65–12.8)	**0**.**003**	9.12 (2.71–38.7)	<0.001
IgG levels (≥8 vs. <8)				
Baseline	0.79 (0.24–3.26)	0.72	–	–
Pre-op	1.92 (0.65–5.25)	0.22	–	–
Peak postop	4.80 (1.61–18.9)	**0**.**004**	3.31 (1.09–11.8)	0.034
IgM levels (≥8 vs. < 8)				
Baseline	1.20 (0.44–4.02)	0.73	–	–
Pre-op	1.43 (0.36–4.36)	0.58	–	–
Peak postop	3.18 (1.29–8.42)	**0**.**011**	–	–
Creatinine ^c^	1.00 (1.00–1.00)	**0**.**041**	–	–

OR, odds ratio; CI, confidence interval; AMR, antibody-mediated rejection; GN/FGS, glomerulonephritis/focal segmental glomerulosclerosis; DM, diabetes mellitus; ESRD, end-stage renal disease; PKE, paired kidney exchange.

Bold values indicate *P* < 0.05.

a Odds ratios and 95% CIs calculated using logistic regression and firth's penalized logistic regression.

b Cluster 1 = Other ABO incompatibility including (A1 to B, A2 to B, B to A, A1B to O); Cluster 2 = anti-O incompatibility (A1/2 to O); Cluster 3 = B to O incompatibility.

c The peak creatinine value within the first postoperative week.

For laboratory parameters, patients with peak postoperative IgG ≥ 8 had a significantly trend toward higher risk of AMR (OR 4.80, 95% CI: 1.61–18.9; *p* = 0.004). Similarly, peak postoperative IgM ≥ 8 was associated with a threefold increased risk of AMR (*p* = 0.011).

### 3.3 Early AMR-free survival and long-term graft and patient outcomes following ABOiKT

**Figure 4:**
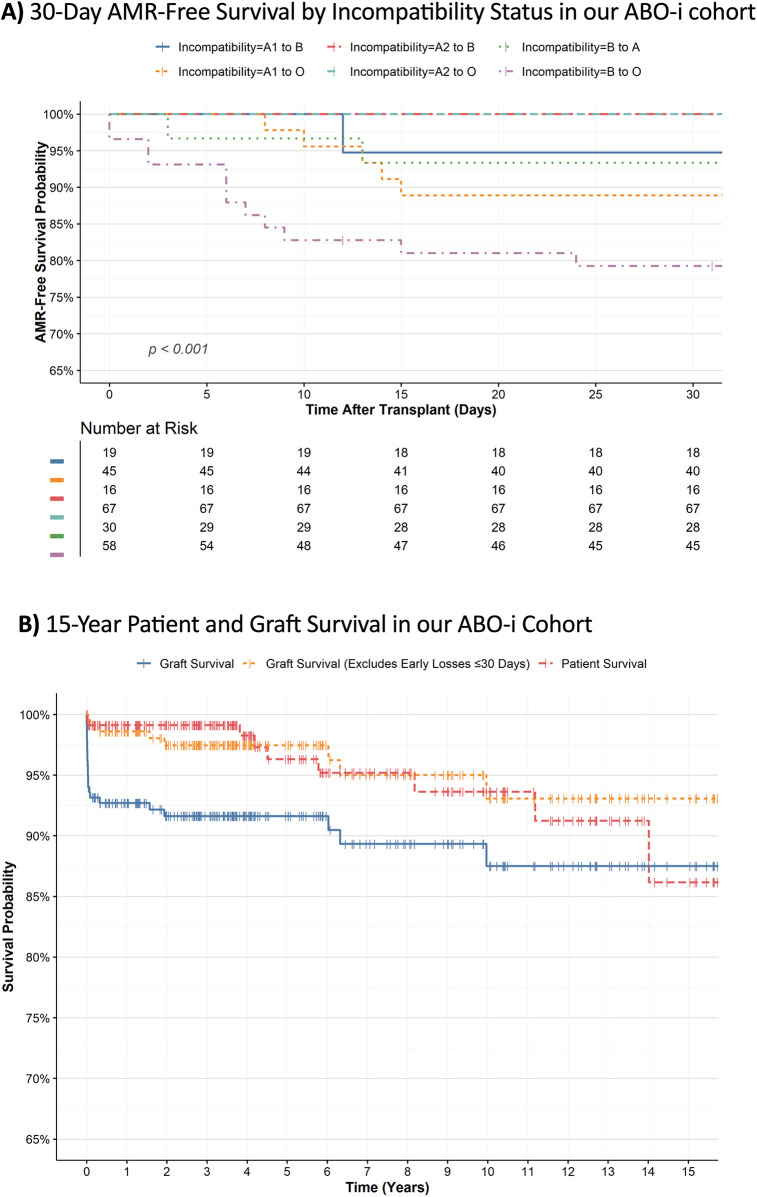
**(A)** Kaplan–Meier survival analysis showing the probability of antibody-mediated rejection (AMR)-free survival over time, stratified by ABO incompatibility type. The bottom table presents the number of patients at risk for each group. **(B)** Fifteen-year survival plot for ABOiKT at our center illustrating the survival probabilities over time for AMR-free (blue solid line), graft (green dashed line), and patient (red dashed line) survival among the transplant recipients. The tick marks represent the censored observations.

Figure 4A presents the Kaplan–Meier estimates of AMR-free survival stratified by ABO incompatibility type, revealing significant differences between blood group incompatibilities (log-rank *p* < 0.001). A2 to O and A2 to B graft recipients maintained 100% AMR-free survival for 30 days. In contrast, B to O incompatibility exhibited the lowest AMR-free survival, 79.2% at 30 days, indicating a higher short-term risk of rejection.

To further evaluate ABOiKT survival, we conducted univariate and multivariate Cox proportional hazards analysis to estimate the risk of graft failure overtime (Table 3). Overall, no significant differences were detected in any of the patients' characteristics tested, except for AMR detection, incompatibility categories, and creatinine levels post operation. After adjusting for AMR and other covariates, B to O incompatibility was associated with a higher risk of graft failure (aHR = 4.47, 95% CI 1.40–0.93, *p* = 0.038). The strong effect of AMR (aHR = 152, 95% CI 25.1–924, *p* < 0.001) largely explained the differences observed in univariate models, highlighting AMR as the dominant determinant of graft loss in our ABOi cohort.

**Table 3:** Univariate cox regression analysis estimating the risk of graft failure by patients’ clinical and demographical data.

Characteristic	Univariate Cox Regression		Multivariate Cox Regression	
	HR (95% CI)*^a^*	*p*-value	aHR (95% CI)^a^	*p*-value
Recipients’ Gender				
Male vs. Female	0.65 (0.28–1.52)	0.32	–	
Recipients’ Age	0.99 (0.96–1.01)	0.34	–	
ESRD				
GN or FSGS vs. DM	2.14 (0.43–10.6)	0.35	–	
Other vs. DM	2.68 (0.52–13.9)	0.24	–	
Unknown vs. DM	2.12 (0.46–9.82)	0.34	–	
AMR	207 (42.1–1,020)	**<0**.**001**	224 (40.2–1,250)	**<0**.**001**
Incompatibility categories				
Other ABO incompatibility vs. Anti-O Incompatibility *^b^*	0.39 (0.11–1.40)	0.15	3.39 (0.93–12.4)	0.064
B to O Incompatibility vs. Anti-O Incompatibility ^b^	6.35 (2.05–19.7)	**0**.**001**	4.47 (1.40–14.3)	**0**.**012**
Baseline IgG ≥8 vs. <8	0.65 (0.17–2.44)	0.52	–	
Pre-op IgG ≥8 vs. <8	1.72 (0.62–4.79)	0.3	–	
Peak postop IgG ≥8 vs. <8	2.90 (0.93–8.99)	0.066	–	
Baseline IgM ≥8 vs. <8	0.89 (0.32–2.43)	0.82	–	
Pre-op IgM ≥8 vs. <8	1.02 (0.30–3.48)	0.97	–	
Peak postop IgM ≥8 vs. <8	1.42 (0.61–3.28)	0.41		
Creatinine *^c^*	1.00 (1.00–1.00)	**<0**.**001**	–	–

HR, hazard ratio; CI, confidence interval; AMR, antibody-mediated rejection; GN/FGS, glomerulonephritis/focal segmental glomerulosclerosis; DM, diabetes mellitus; ESRD, end-stage renal disease; PKE, paired kidney exchange.

Bold values indicate *P* < 0.05.

a Hazard ratios and 95% CIs calculated using cox regression.

b Cluster 1 = Other ABO incompatibility including (A1 to B, A2 to B, B to A, A1B to O); Cluster 2 = anti-O incompatibility (A1/2 to O); Cluster 3 = B to O incompatibility.

c The peak creatinine value within the first postoperative week.

Kaplan–Meier survival analysis showed excellent long-term outcomes after transplantation, particularly among patients without early rejection or graft dysfunction (Figure 4B; Table 4). At 1-month posttransplant, patient survival was 99.5%, while graft survival was 93.6%. Survival rates remained stable over the first year, with patient survival at 99.1% and graft survival at 92.7%. Graft survival excluding early losses remained above 97% during this period. At 15 years posttransplant, patient survival was 86.2%, and graft survival remained at 87.5%, with 93.1% survival in recipients maintaining function beyond the first month.

**Table 4:** Survival-free estimates in patient, graft, and AMR survival for a 15-year follow-up.

Post-Transplant	Patient survival (%)	Graft survival (%)	Graft survival (excluding early losses within 30 days) (%)
1 month	93.6	99.5	99.5
3 months	99.1	93.1	99.1
1 year	99.1	92.7	98.6
3 years	99.1	91.6	97.5
5 years	96.3	91.6	97.5
10 years	93.6	87.5	93.1
15 years	86.2	87.5	93.1

## 4 Discussion

In this study spanning 15 years, we evaluated the outcomes of 239 ABOiKT performed at our center and found excellent patient and graft survival rates. This study was conducted in a setting characterized by a high volume of living-related donors, offering a promising model for expanding donor pools in regions where deceased-donor programs remain limited. Published data on ABOiKT in the Gulf and broader Middle East remain limited, particularly on long-term outcomes. To the best of our knowledge, this study evaluated the largest ABOiKT cohort in the Middle East and is the first to provide long-term outcome data from the region.

Although many studies have examined short-term outcomes, few have reported survival beyond 10 years. In Japan, a large single-center study of 441 ABOiKT recipients reported graft survival rates of 84% at 1 year and 59% at 9 years (13). In contrast, our cohort demonstrated superior outcomes, with graft survival rates of 92.7% at 1 year, 87.5% at 10 years, and 87.5% maintained up to the 15th year. More recent national data from Japan since 2000 indicate 5- and 10-year graft survival rates of 96.7% and 91.8%, respectively, and 96.6% and 89.6% for patient survival, respectively (14). These findings reflect significant progress in ABOiKT management globally and were also observed in our study, highlighting the effectiveness of modern desensitization and immunosuppression protocols in achieving durable graft survival in ABOiKT recipients.

In our cohort, early AMR was observed in 9.2% of the patients and typically within the first 11 days post-transplantation. Despite early rejection events, we maintained satisfactory long-term outcomes. AMR was successfully reversed in a subset of patients (*n* = 8), particularly those with B to O mismatches (4 patients required splenectomy to rescue the grafts), many of whom retained graft function. By 5 years, patient survival remained above 96%, while graft survival exceeded 91%. These findings align with those of a German study with a similar cohort, which reported increased early posttransplant mortality among ABOiTK recipients but favorable long-term outcomes (15). In contrast, a large national cohort study in the United States (2000–2015) reported less favorable outcomes for ABOiKT recipients, showing a significantly higher risk of acute rejection within the first year and a twofold increased risk of death at 1 year compared with ABO-c recipients (4). These disparities underscore the need to investigate regional differences in ABOiKT outcomes.

The global implementation of ABOiKT has evolved as a critical strategy for addressing disparities in organ availability. These regional differences in survival may reflect not only differences in healthcare system infrastructure and immunosuppression protocols but also donor–recipient relationships. In a large UK national registry cohort of 357 ABOiKT recipients, the 5-year graft survival reached 83%, which is lower than the 91% in our cohort, underscoring the efficacy of our standardized desensitization protocol (16).

Building on this success, other European centers have successfully implemented unrelated donor ABOiKT through kidney exchange programs under a standardized protocol, achieving outcomes comparable to those of related donor transplants. For instance, a German study with 137 recipients reported a 15-year follow-up survival rate of 89% and a graft survival rate of 71%, which are comparable to those of ABO-c controls (91% and 87%, respectively), with no significant differences in rejection or infection rates (17). Similarly, a French study found equivalent patient and graft survival rates between ABOi- and ABOc recipients, with comparable rejection rates and allograft function (18). These consistent outcomes across different European countries demonstrate the efficacy of the ABOiKT protocols adopted in the region.

Asia has played a pioneering role in ABOiKT, with Japan among the earliest countries to adopt the approach widely and conducting some of the longest-term outcome studies to date. ABOiKT is well established in Asian countries, with long-term graft survival rates supported by aggressive desensitization protocols and a predominance of living-related donations. Saudi Arabia reports a similar trend, with 68% of our ABOiKT cohort receiving kidneys from related donors. In our study, no significant difference was observed in the incidence of AMR between related and unrelated transplants, a finding consistent with reports from Japan and South Korea observing no significant differences in AMR rates or graft survival between ABO-c and ABO-i transplants in related donors (19, 20).

Interestingly, the degree of ABO incompatibility and associated clinical outcomes varied within our cohort depending on the specific donor and recipient blood subtypes. Transplants involving A2 donors demonstrated 100% rejection-free graft survival over a 15-year period, underscoring the importance of performing blood group A subtyping and supporting the expanded use of A2 donors in ABOiKT programs. This favorable outcome is consistent with existing evidence indicating that A2 to non-A transplants have a lower risk of immunologic complications and markedly decreased expression of A antigens on the renal vascular endothelium (21, 22). In particular, a study with a 10-year follow-up indicated that A2 or A2B to B or O kidney transplants are clinically equivalent to that of ABO-c transplantation (23).

B to O transplants, in contrast, showed higher AMR rates, likely due to the immunological burden from naturally occurring anti-A and anti-B IgG antibodies in group O recipients and the increased endothelial expression of B antigens. Therefore, O recipients are predisposed to stronger antibody mediated cytotoxic responses, increasing the risk of rejection upon exposure to these antigens. Furthermore, the variability in individual desensitization responses and innate immune activation may further contribute to the increased risk of AMR (24, 25). All these factors increase the risk of this group to rejection (13, 26). Moreover, evidence in the literature supporting these findings is limited, as many ABOi studies evaluated overall survival without considering blood group incompatibilities.

One promising therapeutic approach to improve the outcomes of B to O transplants and reduce the risk of AMR is enzymatic conversion. A recent preclinical study successfully transplanted B-zyme-treated kidneys into type O brain-dead recipients, demonstrating good tolerance without signs of AMR. However, this strategy is still in its experimental phase, requiring further investigations to validate its clinical safety and efficacy (27). Overall, these findings highlight the need to consider blood subgroup variations when evaluating the risks and feasibility of ABOiKT.

In this study, we have reported IgG and IgM anti-ABO antibody titers at three major time points: before desensitization, on the morning of transplantation, and in the early posttransplant phase. Notably, only the posttransplant IgG and IgM levels were predictive of AMR, whereas baseline titers and those measured on the day of surgery did not demonstrate an association with AMR occurrence. This observation is consistent with previous reports demonstrating the association between elevated posttransplant titers and an increased risk of AMR (28, 29). Although the dynamics of IgG and IgM titers in the pretransplant period remain clinically relevant, emerging evidence indicates that these antibody titers are not equivalent in terms of predictive value. One study reported that high pretransplant IgM titers, even when IgG levels were low, were strongly associated with early AMR and thrombotic microangiopathy after kidney transplantation (30). However, in our cohort, pretransplant IgG and IgM titers demonstrated limited predictive value. In contrast, posttransplant antibody measurements were associated with AMR, consistent with the results of previous studies (30–32). Overall, these findings support the growing emphasis on posttransplant immunological surveillance rather than reliance on pretransplant titers alone.

This study has several limitations, including its retrospective, single-center design, which may limit the generalizability of our findings to other settings. The predominance of young, related living donor transplants further constrains the broader applicability of the results. Another limitation of our study is the exclusion of ABOc. A comparative analysis between the ABOi and ABOc cohorts will be conducted in our future work to evaluate whether outcomes differ after adjusting for baseline donor and recipient characteristics.

The study's strengths include its large, well-characterized cohort with a 15-year follow-up, making it one of the most comprehensive ABOiKT studies. Detailed analysis of ABO mismatch subtypes, meticulous desensitization and immunosuppressive protocols, and clear reporting of both short- and long-term patient and graft survival outcomes provide valuable global and regional insights. Importantly, our study benefits from being conducted in a Middle Eastern population, where blood group B is more prevalent than in Western countries. A recent study has shown that approximately, 26% of the Saudi population has blood group B (33). In Comparison only 9% of the population the in the US has blood group B (34). This demographic feature provided a unique opportunity to examine a relatively large number of ABO-i transplants from B donors to O recipients, a combination that is comparatively rare in European and North American cohorts (34). Consequently, our findings help fill a critical gap in the literature, where most prior studies were underpowered to detect associations specific to (B to O) incompatibility.

In conclusion, this study presents compelling evidence for the long-term safety and efficacy of ABOiKT, as acquired from cases treated at our center, demonstrating favorable outcomes across different mismatch types. Although initial graft losses were higher in the B to O and A1 to O mismatched pairs, the exceptional 15-year patient and graft survival rates demonstrate the durability of these transplants. Notably, the superior performance of A2 donor transplants, all of which achieved 100% rejection-free graft survival, has reshaped our program's strategy to prioritize this subgroup. As the largest study on ABOiKT in the Middle East with extended follow-up, our work offers valuable regional and global insights and significantly enhances the global understanding of ABOiKT outcomes.

## Data Availability

The datasets presented in this study can be found in online repositories. The names of the repository/repositories and accession number(s) can be found in the article/Supplementary Material.
