# Supplementary material for: Impact of different blood group incompatibilities in kidney transplantation: a 15-year outcomes analysis from a large kidney transplant center
Source: Front Transplant. 2025 Nov 18;4:1690999. doi: 10.3389/frtra.2025.1690999 (PMC12669197; doi:10.3389/frtra.2025.1690999)
Supplement: Supplementary file 1 [file Table1.docx]

**Appendix**

**Desensitization protocol for ABOi-KT**
Desensitization prior to ABOi-KT consisted of rituximab, plasmapheresis, and intravenous immunoglobulin (IVIG). A single 500-mg dose of rituximab was administered within 2 weeks before transplantation. Therapeutic plasma exchange (TPE) using **5% or 10% albumin** was conducted preoperatively, with the number of sessions tailored to each patient’s anti-A/B antibody titers. **In cases with bleeding risks or coagulation abnormalities, albumin was replaced with fresh-frozen plasma (FFP). The FFPs used were either of the donor’s blood group or type AB.** Each session of plasmapheresis was followed by IVIG at a dose of 100 mg/kg. Additional post-transplant plasmapheresis was administered only when a significant rebound in the antibody titers occurred or a clinical impression of AMR was noted.

In the majority of participants, ABO antibody levels were assessed using the **tube agglutination method**, a semiquantitative technique used to evaluate both (IgM) and (IgG) isoagglutinins. The tube method involves performing serial twofold dilutions of patient serum combined with red cells of known antigenicity. The degree of agglutination at each dilution was visually scored and summed. The antibody titer provides an estimate of the circulating antibody burden and was reported as the reciprocal of the highest dilution exhibiting 1+ visible agglutination. After the surgery, the antibody titers were monitored daily for 2 weeks postoperatively after which the monitoring was discontinued.

Prior to July 2013, monitoring was limited to IgM antibody titers. Thereafter, both IgM and IgG titers were routinely measured, with target pretransplant thresholds of ≤1:8 for IgM and ≤1:16 for IgG.

Immunosuppressive therapy consisted of induction with rabbit anti-thymocyte globulin (thymoglobulin) and maintenance with a triple-drug regimen of tacrolimus, mycophenolate mofetil, and corticosteroids. Thymoglobulin was administered at a dose of 1.5 mg/kg/day for 3 days consecutively. The fourth dose was administered if the absolute CD3 count was above 20 mm^3^. Mycophenolate mofetil was initiated 24 hours before transplantation, while tacrolimus was started postoperatively once adequate urine output was confirmed. Trough levels of tacrolimus were maintained between 8 and 10 ng/mL for the first 3 months post-transplant and then adjusted to 6–8 ng/mL thereafter.

Patients received trimethoprim-sulfamethoxazole (480 mg daily) prophylaxis against *Pneumocystis* infection and against cytomegalovirus with valganciclovir (450 mg daily, adjusted for kidney function). We routinely administered IVIG 2 weeks after transplantation, up to a total dose of 2 g/kg, including the doses required after each session of TPE.

Perioperative antibiotic coverage was administered to reduce the risk of bacterial infections. Up until June 2013, piperacillin/tazobactam (2.25 g three times daily) was administered. Following updates to hospital guidelines, this was replaced with cefoxitin (1 g/day).

**Table S1.** Isohemagglutinin titers of IgM and IgG across three timepoints by AMR Status at 30 days. Values represent the mean (SD). P-values are based on the Wilcoxon rank sum test.

|  | Baseline | | | Morning of the Surgery | | | Maximum | | |
| --- | --- | --- | --- | --- | --- | --- | --- | --- | --- |
|  | **No AMR** N = 148*^1^* | **AMR** N = 13*^1^* | **p-value***^2^* | **No AMR** N = 174*^1^* | **AMR** N = 16*^1^* | **p-value***^2^* | **No AMR** N = 152*^1^* | **AMR** N = 17*^1^* | **p-value***^2^* |
| IgG Titer Category |  |  | 0.7 |  |  | 0.5 |  |  | 0.035* |
| 0 | 2 (1.4%) | 0 (0.0%) |  | 11 (6.3%) | 0 (0.0%) |  | 0 (0.0%) | 0 (0.0%) |  |
| 1 | 6 (4.1%) | 0 (0.0%) |  | 23 (13.2%) | 0 (0.0%) |  | 0 (0.0%) | 0 (0.0%) |  |
| 2 | 7 (4.8%) | 1 (7.7%) |  | 37 (21.3%) | 4 (25.0%) |  | 30 (19.7%) | 1 (5.9%) |  |
| 4 | 13 (8.8%) | 2 (15.4%) |  | 64 (36.8%) | 6 (37.5%) |  | 39 (25.7%) | 2 (11.8%) |  |
| 8 | 36 (24.5%) | 2 (15.4%) |  | 26 (14.9%) | 5 (31.3%) |  | 40 (26.3%) | 4 (23.5%) |  |
| 16 | 24 (16.3%) | 2 (15.4%) |  | 8 (4.6%) | 1 (6.3%) |  | 21 (13.8%) | 4 (23.5%) |  |
| 32 | 33 (22.4%) | 6 (46.2%) |  | 4 (2.3%) | 0 (0.0%) |  | 16 (10.5%) | 2 (11.8%) |  |
| 64 | 18 (12.2%) | 0 (0.0%) |  | 1 (0.6%) | 0 (0.0%) |  | 5 (3.3%) | 3 (17.6%) |  |
| 128 | 5 (3.4%) | 0 (0.0%) |  | 0 (0.0%) | 0 (0.0%) |  | 0 (0.0%) | 0 (0.0%) |  |
| 256 | 3 (2.0%) | 0 (0.0%) |  | 0 (0.0%) | 0 (0.0%) |  | 0 (0.0%) | 0 (0.0%) |  |
| 512 | 0 (0.0%) | 0 (0.0%) |  | 0 (0.0%) | 0 (0.0%) |  | 1 (0.7%) | 1 (5.9%) |  |
|  | **No AMR** N = 218*^1^* | **AMR** N = 20*^1^* | **p-value***^2^* | **No AMR** N = 218*^1^* | **AMR** N = 20*^1^* | **p-value***^2^* | **No AMR** N = 218*^1^* | **AMR** N = 20*^1^* | **p-value***^2^* |
| Igm Titer Category |  |  | 0.7 |  |  | 0.5 |  |  | 0.003* |
| 0 | 2 (0.9%) | 0 (0.0%) |  | 17 (7.8%) | 0 (0.0%) |  | 0 (0.0%) | 0 (0.0%) |  |
| 1 | 5 (2.3%) | 0 (0.0%) |  | 45 (20.6%) | 2 (10.0%) |  | 0 (0.0%) | 0 (0.0%) |  |
| 2 | 10 (4.6%) | 1 (5.0%) |  | 60 (27.5%) | 8 (40.0%) |  | 50 (26.7%) | 3 (15.0%) |  |
| 4 | 34 (15.6%) | 3 (15.0%) |  | 71 (32.6%) | 7 (35.0%) |  | 55 (29.4%) | 4 (20.0%) |  |
| 8 | 36 (16.5%) | 6 (30.0%) |  | 22 (10.1%) | 3 (15.0%) |  | 45 (24.1%) | 3 (15.0%) |  |
| 16 | 52 (23.9%) | 5 (25.0%) |  | 3 (1.4%) | 0 (0.0%) |  | 24 (12.8%) | 3 (15.0%) |  |
| 32 | 37 (17.0%) | 4 (20.0%) |  | 0 (0.0%) | 0 (0.0%) |  | 10 (5.3%) | 3 (15.0%) |  |
| 64 | 26 (11.9%) | 0 (0.0%) |  | 0 (0.0%) | 0 (0.0%) |  | 2 (1.1%) | 1 (5.0%) |  |
| 128 | 15 (6.9%) | 1 (5.0%) |  | 0 (0.0%) | 0 (0.0%) |  | 1 (0.5%) | 1 (5.0%) |  |
| 256 | 1 (0.5%) | 0 (0.0%) |  | 0 (0.0%) | 0 (0.0%) |  | 0 (0.0%) | 0 (0.0%) |  |
| 512 | 0 (0.0%) | 0 (0.0%) |  | 0 (0.0%) | 0 (0.0%) |  | 0 (0.0%) | 2 (10.0%) |  |
| *^1^* n (%) | | | | | | | | | |
| *^2^* Fisher’s exact test | | | | | | | | | |

**Table S2.** Line graph for Serum Creatinine (μmol/L) levels at seven time points across different ABO mismatch groups.

|  | **A1 to B^1^** | **A1 to O^1^** | **A2 to B^1^** | **A2 to O^1^** | **B to A^1^** | **B to O^1^** | **P-value^2^** |
| --- | --- | --- | --- | --- | --- | --- | --- |
| **Day 5** | 121.0 (113.2) | 98.5 (154.6) | 88.5 (91.7) | 118.0 (169.9) | 118.0 (177.6) | 113.0 (156.4) | 0.84 |
| **Day 30** | 102.5 (28.7) | 99.0 (48.3) | 94.5 (50.6) | 95.0 (53.4) | 94.0 (148.0) | 88.0 (79.9) | 0.75 |
| **3 Months** | 97.5 (20.9) | 98.0 (41.1) | 92.0 (22.7) | 90.0 (30.8) | 91.5 (35.5) | 84.0 (39.7) | 0.26 |
| **6 Months** | 97.0 (16.3) | 97.0 (35.6) | 94.0 (17.8) | 89.0 (31.7) | 97.0 (80.2) | 88.0 (35.9) | 0.45 |
| **1 Year** | 91.0 (17.8) | 92.5 (27.9) | 89.0 (13.6) | 79.0 (25.1) | 84.0 (87.5) | 86.0 (54.7) | 0.75 |
| **3 Years** | 96.5 (23.4) | 81.0 (39.6) | 82.0 (21.4) | 87.0 (21.5) | 84.0 (184.4) | 79.0 (133.4) | 0.8 |
| **5 Years** | 96.0 (11.6) | 88.0 (63.9) | 79.0 (15.8) | 88.0 (25.2) | 80.0 (37.9) | 89.5 (19.5) | 0.75 |
| **10 Years** | 94.5(262.7) | 79(36.9) | 95.5(4.9) | 86.5(30.2) | 110.0(50.8) | 85.0(189.3) | 0.84 |
| **15 Years** | 105.5(10.61) | 61.0(129.1) | 116.0(NA) | 86.0(38.2) | NA | 88(51.3) | 0.75 |
| **p-value^1^** | 0.28 | 0.07 | 0.61 | <0.001* | 0.2 | 0.06 |  |

^1^ Data are shown in median (standard deviation), ^2^ Kruskal-Wallis rank sum test
